# Supplementary material for: Conserved roles for the dynein intermediate chain and Ndel1 in assembly and activation of dynein
Source: Nat Commun. 2023 Sep 20;14:5833. doi: 10.1038/s41467-023-41466-5 (PMC10511499; doi:10.1038/s41467-023-41466-5)
Supplement: Supplementary file 1 — Supplementary Information [file 41467_2023_41466_MOESM1_ESM.pdf]

Conserved Roles for the Dynein Intermediate Chain and Ndel1 in Assembly and Activation of Dynein

Kyoko Okada[1,‡], Bharat R. Iyer[2,‡], Lindsay G. Lammers[2], Pedro A. Gutierrez[1,3], Wenzhe Li[1], Steven M. Markus[2,#] and Richard J. McKenney[1,#]

Supplementary Information

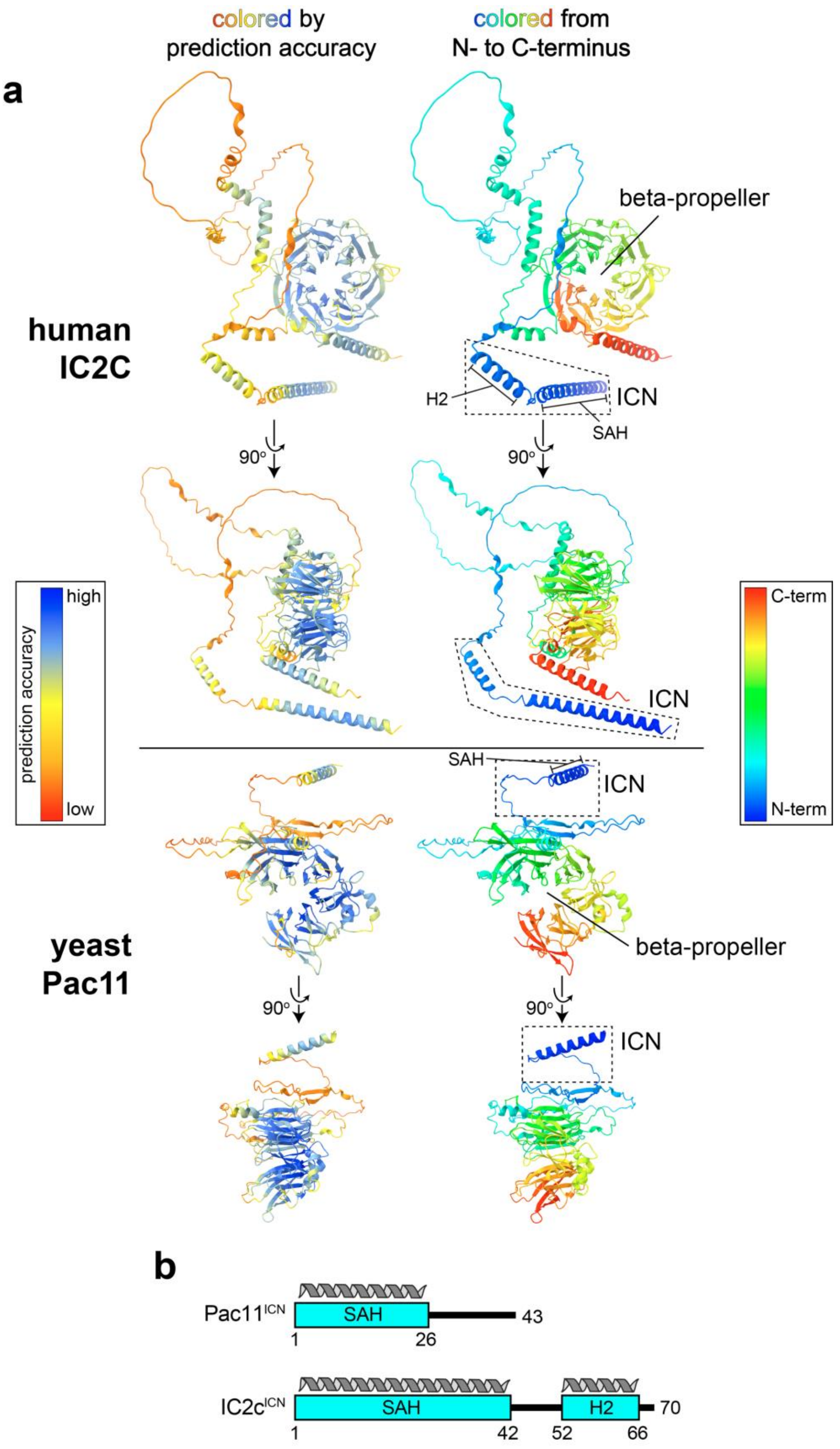

Supplementary Figure 1. AlphaFold2-generated models of dynein intermediate chains from human and yeast. **a** AF2 Models are colored according to prediction accuracy (left) or from N- to C-terminus (right). **b** Schematic of yeast and human intermediate chain N-termini deleted in this study (SAH, single alpha helix; H2, helix 2).

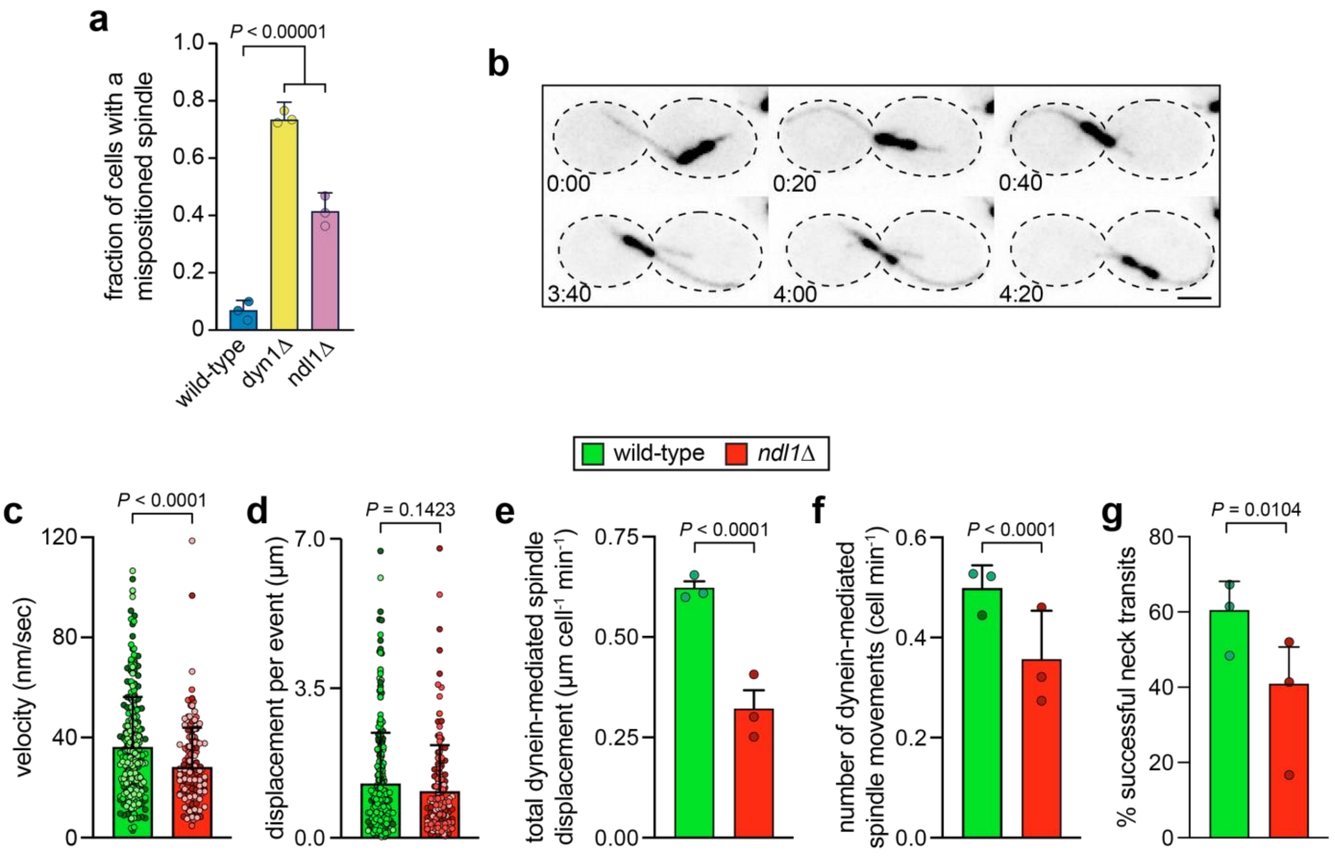

Supplementary Figure 2. Quantitative assessment of Ndl1's role in cellular dynein function. **a** Plot (weighted mean ± weighted standard error of proportion, along with values from individual replicates) depicting fractions of cells exhibiting mispositioned anaphase spindles (n = 53/56/49 WT cells, 38/56/61 *dyn1Δ* cells, and 62/61/55 *ndl1Δ* cells, all from 3 independent replicates). Two-tailed *P* values were generated by calculating Z scores. **b** Representative time-lapse inverse fluorescence images of a hydroxyurea (HU)-arrested *kar9Δ* cell (*KAR9* was deleted to more reliably score dynein-dependent spindle movements) exhibiting typical dynein-mediated spindle movements (scale bar, 2 μm). **c - g** Plots of indicated parameters for spindle dynamics in *kar9Δ NDL1* and *kar9Δ ndl1Δ* strains. Briefly, the mitotic spindles were tracked in 3-dimensions using a custom written Matlab code[63,64]. Dynein-mediated spindle movements were manually selected from the tracking data, from which velocity **c**, displacement (**d**, per event; or, **e**, per minute), and the number of dynein-mediated spindle movements per minute **f** were obtained. The fraction of successful neck transits (**g**; successful attempts divided by total attempts) were manually scored. Each bar represents the mean ± SD (shown along with the means from each independent replicate; n = 20/20/20 and 19/17/20 HU-arrested WT and *ndl1Δ* cells, respectively, from 3 independent replicates). For panels **c - g**, *P* values were calculated using a Mann-Whitney test or a two-tailed unpaired Welch's t-test.

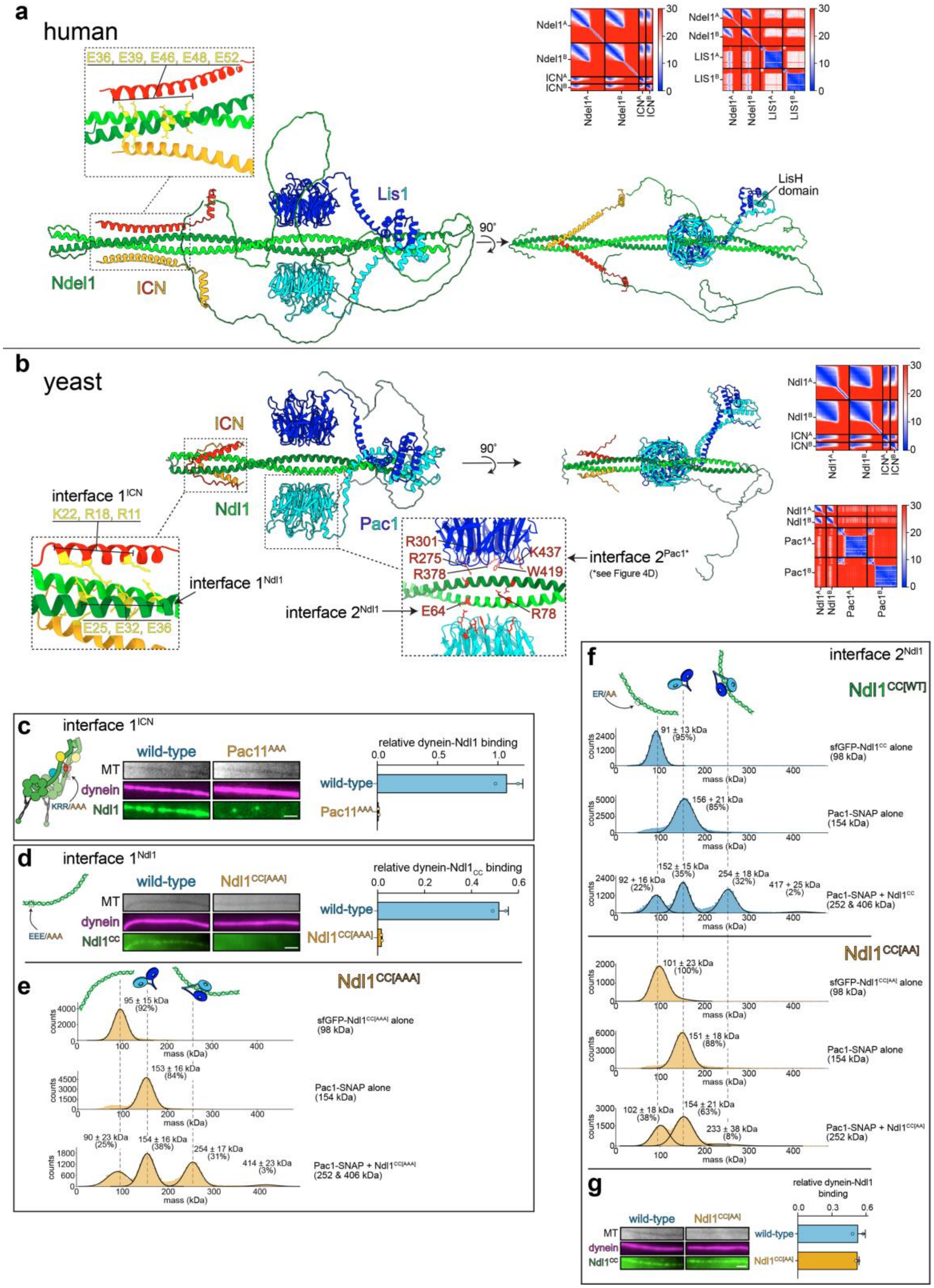

Supplementary Figure 3. AlphaFold2-Multimer predictions of Ndel1/Ndl1-LIS1/Pac1-ICN complex structures, and validation thereof. **a** Model of Ndel1-LIS1-ICN complex (dimers of each) along with predicted alignment error (PAE) plots. Note that models of Ndel1-LIS1 and Ndel1-ICN were generated independently, and assembled into a co-complex using ChimeraX. Inset shows residues on Ndel1 previously shown to be important for interaction with dynein[63]. **b** Model of Ndl1-Pac1-ICN complex (dimers of each) along with PAE plots. Note that models of Ndl1-Pac1 and Ndl1-ICN were generated independently and assembled into a co-complex using ChimeraX. Insets show residues on Ndl1, Pac1 and ICN predicted by AF2 models that would be important for contacts at interface 1 (ICN-Ndl1) and interface 2 (Pac1-Ndl1). **c and d** Representative images from TIRFM binding assay and plots (mean ± SD, along with mean values from independent replicates) depicting the effect of mutating interface 1 on either Pac11 (R11A, R18A, K22A; in the context of the full-length dynein complex purified from yeast) or Ndl1^{CC} (E25A, E32A,

E36A) on the Ndl1-dynein interaction (n = 20 MTs for each data point from 2 independent replicates). **e** Mass photometric analysis of individual proteins, or mixtures of proteins, as indicated. Note that Ndl1$^{CC[AAA]}$, which doesn't bind to dynein, is competent for interaction with Pac1 (apparent from 254 kDa species). **f** Mass photometric analysis of individual proteins, or mixtures of proteins, as indicated, depicting the effect of mutating interface 2 on Ndl1^{CC} (E64A, R78A) on its interaction with Pac1. Note that Ndl1$^{CC[AA]}$ cannot bind Pac1, as is apparent from lack of 254 kDa species. **G** Representative images from TIRFM binding assay and plot (mean ± SD, along with mean values from independent replicates) depicting the ability of Ndl1$^{CC[AA]}$ to interact with dynein (n = 20 MTs for each data point from 2 independent replicates). For mass photometry, equal volumes of prediluted proteins (100 nM Ndl1 and 200 nM Pac1) were first mixed together, and then diluted 5-fold in assay buffer on the stage immediately prior to imaging (for final concentrations of 10 nM Ndl1 and 20 nM Pac1). Assessments of individual proteins were performed by diluting prediluted proteins alone 10-fold in assay buffer prior to imaging. All scale bars, 2 μm.

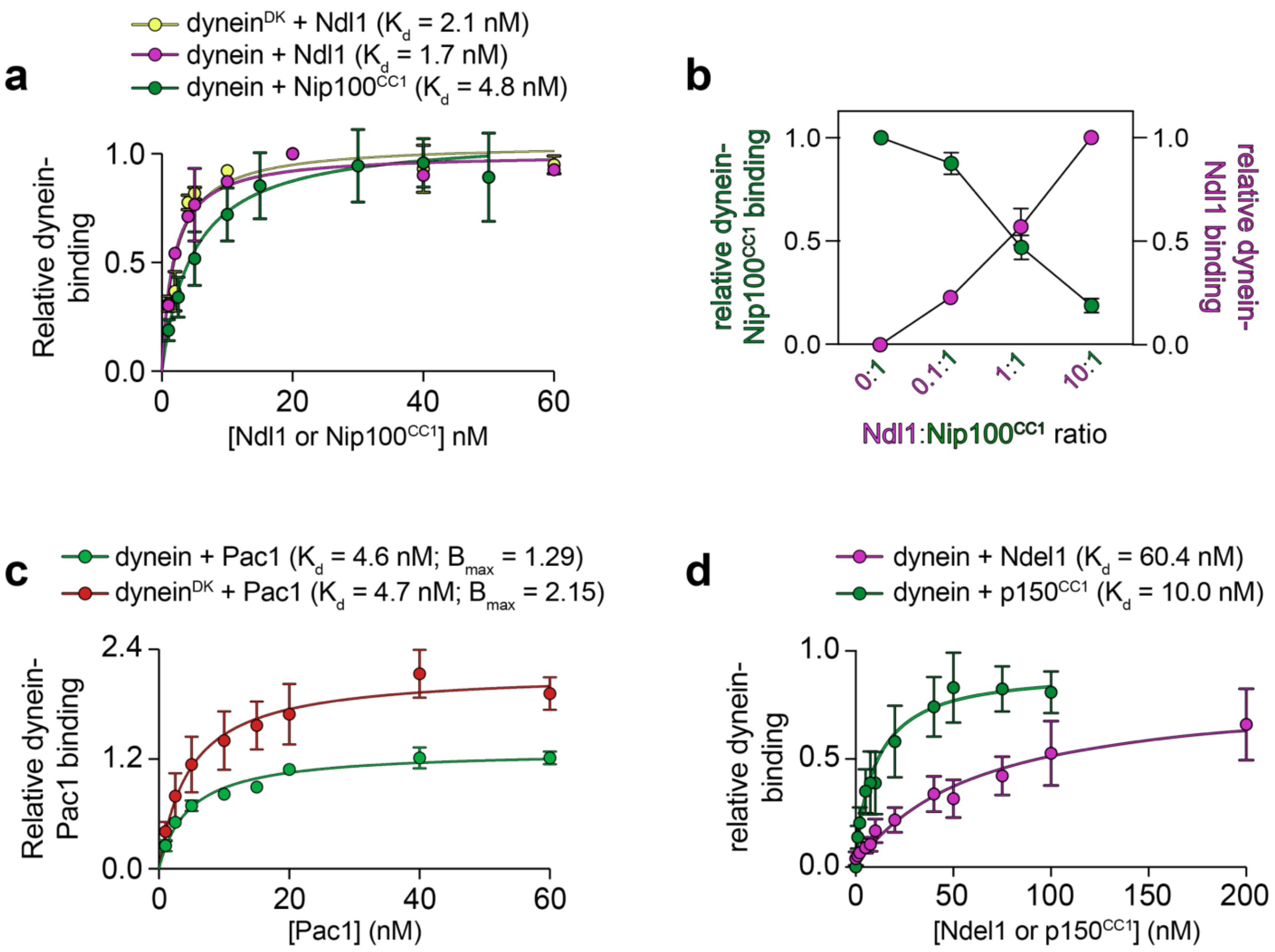

Supplementary Figure 4. Relative binding affinities of dynein for p150^{CC1}/Nip100^{CC1} and Ndel1/Ndl1 . a Plot depicting relative dynein-Ndl1 (wild-type, or dynein^{DK}, a mutant predicted to be unable to adopt the phi conformation with D2868K substitution[62]) or dynein-Nip100^{CC1} binding, as determined by TIRFM-based assay. The full-length dynein complex (wild-type or dynein^{DK}, as indicated; both purified from yeast) was incubated with indicated concentrations of either Ndl1 or Nip100^{CC1}, and introduced into an imaging chamber with coverslip-immobilized microtubules. Relative binding at indicated concentrations of Ndl1 and Nip100^{CC1} was measured (determined from background corrected microtubule-localized intensities of Ndl1-SNAPf^{JFX554} divided by that of HALO^{JF503}-dynein, or eGFP-Nip100^{CC1} divided by that of HALO^{JFX549}-dynein), and then plotted and fitted to binding curves using Graphpad Prism. Each point represents the mean ± SD (n = 20 MTs for each data point from 2 independent replicates). **b** Plot depicting relative dynein-Ndl1 and dynein-Nip100^{CC1} binding (determined as in panel **a**) for indicated ratio of Ndl1:Nip100^{CC1}. For each datapoint, a fixed concentration of full-length dynein was incubated with a mixture of Ndl1 and Nip100^{CC1}, introduced into an imaging chamber with coverslip-immobilized microtubules, and immediately imaged. Each point represents the mean ± SD (n = 20 MTs for each data point from 2 independent replicates). **c** Plot depicting relative Pac1 binding by dynein (wild-type, or dynein^{DK}, as indicated; determined as in panel **a**) for indicated concentration of Pac1. Each point represents the mean ± SD (n = 20 MTs for each data point from 2 independent replicates). **d** Plot depicting relative dynein-Ndel1 or dynein-p150^{CC1} binding, as determined by TIRFM-based assay. The full-length human dynein complex was incubated with indicated concentrations of either Ndel1 or p150^{CC1}, and introduced into an imaging chamber with coverslip-immobilized microtubules. Relative binding was measured as above in panel **a**. Each point represents the mean ± SD (n = 59/51, 61/53, 55/55, 52/51, 61/56, 61/57, 62/50, 81/57, 67/53, 76/55, 64/55, 64/55, 51/51 for Ndel1, 59/59, 56/60, 60/54, 61/58, 53/56, 90/71, 59/66, 90/64, 85/57, 54/59, 56/56 for p150^{CC1} from two independent replicates). Each point represents the mean ± SD.

Supplementary Table 1. Oligos used in this study.

Oligo #	Purpose	Direction	Primer sequence
For yeast constructs:			
1	To truncate Pac11 in p8His-ZZ-HALO-Dynein complex plasmid (deleting residues 1-43). PCR from p8His-ZZ-HALO-Dynein with P2, and assemble into same digested with KpnI/KasI along with PCR products from P3 + P4 and P5 + P6.	forward	TAAAGGGAACAAAAGCTGGGTAC
2	See P1 for details.	reverse	TCTGTGCTGTTGATTGTGGATTCATT C
3	See P1 for details.	forward	GAATCCACAATCAACAGCACAGAGAC
4	See P1 for details.	reverse	CCTCCTTGACGTTAAAGTATAGAGGT ATATTAAC
5	See P1 for details.	forward	TCTATACTTTAACGTCAAGGAGG ATG GCAACCATGGTCAGCGTTTC
6	See P1 for details.	reverse	TACTGGCCCCCCTAACAAC
7	To add S6 tag (GDSLSWLLRLLN) at the C-terminus of Dyn2. PCR with P8 from p8His-ZZ-HALO-Dynein *or* p8His-ZZ-HALO-Dynein[Pac11$^{\Delta ICN}$] and assemble into the same, each digested with KasI/KpnI along with PCR products from P9 & P10 and P11 & P12.	forward	CACTAAAGGGAAACAAAAGCTGG
8	See P7 for details.	reverse	CTAGTTTAATAGCCTAAGCAGCCAGG AAAGGCTGTCTCCGGAGGCGGAGCC TGCTGTTTTGAAAACTAAAAACGCC
9	See P7 for details.	forward	GGCTGCTTAGGCTATTAAACTAGGCT CTAGATATATATATATATATATATAT AACTGTCTAGAAATAAAGAGTATC
10	See P7 for details.	reverse	GGGGAATATATGATTGCTGTTATCGT AG
11	See P7 for details.	forward	ATAACAGCAATCATATATTCCCCATC AAG
12	See P7 for details.	reverse	TACTGGCCCCCCTAACAAC
13	To clone Ndl1 coiled-coil (residues 2-132) into pET28a:6His-StrepII-sfGFP). PCR with P14 and assemble into pET28a:strepII-sfGFP-Spindly[1-316] (from ref. 12) digested with KpnI and NotI.	forward	TACAAAGGTGCTGGAGCAGGTAC C GTGCCGAATTTGGATTTGGAAAC
14	See P13 for details.	reverse	TGGTGGTGGTGGTGCTCGAGTGC GGCCGC CTA GGGTAACGACCTGGTGTTC

15	To clone Nip100 coiled-coil (residues 97-377) into pGEX-KG:SUMO. PCR with P16 from yeast genomic DNA and assemble into pGEX-KG:SUMO digested with NheI and HindIII.	forward	ACAGATTGGTGGTGCCGCCGCCG CTAGC GAGCATAGTTTGCTAAATGGTAATGC AG
16	See P15 for details.	reverse	CGTCAGTCAGTCACGATGAATTA AGCTT CTA TTGATATAATTCCTCCTGTAAAGTGT TCAC
17	To add EGFP to pGEX-KG:SUMO-Nip100-CC. PCR With P18 from pFA6a-GFP(S65T)-TRP1 and assemble into pGEX-KG:SUMO-Nip100-CC digested with NheI.	forward	ACAGATTGGTGGTGCCGCCGCCG CTAGC ATG GTC AGTAAAGGAGAAGAACTTTTCACTG G
18	See P17 for details.	reverse	TACCATTTAGCAAACTATGCTCG CT ACC ACT ACC GCT ACC TTTGTATAGTTCATCCATGCCATGTG
19	To generate pRS306:EGFP-Nip100-CC. PCR with P20 from yeast genomic DNA and assemble into pRS306:GAL1p:EGFP digested with BamHI/NotI.	forward	ACTATACAAAGGTTCTGGAAGTG GATCC GAGCATAGTTTGCTAAATGGTAATGC AG
20	See P19 for details.	reverse	AATTGGAGCTCCACCGCGGTGGC GGCCGC CTA TTGATATAATTCCTCCTGTAAAGTGT TCAC
21	To mutate Pac11 (R11A, R18A and K22A). PCR from p8His-ZZ-HALO-Dynein with P2 and assemble into same vector digested with KpnI and KasI along with PCR products from P3 + P22 and P23 + P6.	forward	TAAAGGGAAACAAAAGCTGGGTAC
22	See P21 for details.	reverse	AGC TCGCCTCTC AGC CAGCTCTTTCAATTGTCT AGC TTTTTCCTCCAGTTGCTTCAATC
23	See P21 for details.	forward	AAGAGCTGGCTGAGAGGCGAGCT CAAGCCAGCTTGTTCCCTG
24	To mutate pRS306:GAL1p:Ndl1-FLAG-SNAP-TEV-ZZ (E25A, E32A, E36A). PCR from pRS306:GAL1p:Ndl1-FLAG-SNAP-TEV-ZZ with P25 and assemble into same vector digested with KpnI and MfeI along with PCR product from P26 + P27.	forward	TACTTTAACGTCAAGGAGGGTAC
25	See P24 for details.	reverse	AGC CAAATCATT AGC ATATTCTTTCGTGGCGCC AGC AAGCTCGCTCAAC
26	See P24 for details.	forward	AAGAATATGCTAATGATTTGGCT CAAGTAATATCAAAGTTAAAAAGCGA TCTTC

27	See P24 for details.	reverse	GTTCCAGTCGTCGGTCACTCTCC
28	To mutate pRS306:GAL1p:8xHIS-ZZ-TEV-Pac1-FLAG-SNAPf ("5a", or R275A, R301A, R378A, W419A, K437A). PCR from pRS306:GAL1p:8xHIS-ZZ-TEV-Pac1-FLAG-SNAPf with P29 and assemble into same vector digested with EcoRI and NotI along with PCR products from P30 + P30 and P31+ P32.	forward	GCAAGAATCAGTGGACCGG
29	See P28 for details.	reverse	CGACCAACCATTGTGGAAGTCCCAAA TTTTAACAGTTTGATC AGC GGAACAGGACGCTATATGG
30	See P28 for details.	forward	GGGACTTCCACAATGGTTGGTCGTTG AAAACATTTCAGCCTCATTCCCAATG GGTT GCT TCCATAGACGTTCTAGGCG
31	See P28 for details.	reverse	ATCATCAGCACAAGAAAACAAGT
32	See P28 for details.	forward	ACTTGTTTTCTTGTGCTGATGAT GCT TCTGTCAGATGTTGGGATC
33	To mutate pRS306:GAL1p:Ndl1-FLAG-SNAP-TEV-ZZ (E25A, E32A, E36A). PCR from pRS306:GAL1p:Ndl1-FLAG-SNAP-TEV-ZZ with P34 and assemble into same vector digested with KpnI and MfeI along with PCR product from P35 + P36.	forward	TACTTTAACGTCAAGGAGGGTAC
34	See P33 for details.	reverse	AGC CAAATCATT AGC ATATTCTTTCGTGGCGCC AGC AAGCTCGCTCAAC
35	See P33 for details.	forward	AAGAATATGCTAATGATTTGGCT CAAGTAATATCAAAGTTAAAAAGCGA TCTTC
36	See P33 for details.	reverse	GTTCCAGTCGTCGGTCACTCTCC
For metazoan constructs:			
CP-10	To amplify pET28a backbone	forward	GATTACAAGGATGACGACGA
CP-11	To amplify pET28a backbone	reverse	ACTTCCAGGTCCTTGGAAGAGAACCT CAAGGGATCCTTTGTAGAGCTCAT
KO001	To amplify Ndel1	forward	TCTTCCAAGGACCTGGAAGTATGGAT GGTGAAGATATACCAG
KO002	To amplify Ndel1	reverse	TCGTCATCCTTGTAATCACCTGATCC AGACACACTGAGAGGTAGCATAC
KO003	To generate Ndel1 E119A	forward	GCATAAGTATGTGAGAGAGCTGGCG CAGGCCAACGACGAC
KO004	To generate Ndel1 E119A	reverse	GTCGTCGTTGGCCTGCGCCAGCTCTC TCACATACTTATGC
KO005	To generate Ndel1 R130A	forward	GACCTGGAGCGAGCCAAAGCGGCAA CAATAGTTTCACTGG

KO006	Quikchange primer to generate Ndel1 R130A	reverse	CCAGTGAAACTATTGTTGCCGCTTTG GCTCGCTCCAGGTC
KO010	To amplify Ndel1-CC	reverse	TTATCGTCATCCTTGTAATCACCTGA TCCAGAAGCCGACTTTCTAGTTACTT C
KO063	To amplify IC2C	forward	TCACGCGTACTAGTGCCACCATGTCA GACAAAAGTGAATTAAAAGCTGAGT TG
KO064	To amplify IC2C	reverse	CCGCCACCCATATGAGATCCACCTCC ACC GGCAGGAATCCGGGTAGC
KO065	To amplify purification tag	forward	GGATCTCATATGGGTGGCGGATC
KO066	To amplify purification tag	reverse	CTTGTCATCGTCATCCTTGTAGTCGC
KO068	To generate ΔICN	forward	TCACGCGTACTAGTGCCACCATGACA GACTCCCCAATTGTCCCTC
KO134	gBlock to append Prescission-tag and myc-tag to Hook3 1-552	-	CTTGAGGTGCTTTTCCAGGGGCCAG GTTCCGGCTCTTTAGAAGTATTGTTC CAAGGTCCGGGGTCAGGTAGTGAAC AAAAACTTATCAGCGAAGAAGATTTA GGCTCAGGATCAGAGCAAAAATTAA TCAGCGAGGAGGATCTGGGAAGTGG GTCTGAGCAAAAGCTTATCTCAGAGG AAGACTTAGGGAGTGGATCT
KO135	To amplify pET28a-Hook3 1-552	reverse	GACTTAGGGAGTGGATCTATGGACA AAGACTGCGAAATGAAG
KO136	To amplify pET28a-Hook3 1-552	forward	CTGGAAAAGCACCTCAA GACTGCCTTTTTCGAACTGCG
KO329	To amplify p150-CC1	forward	AGGTTCTCTTCCAAGGACCTGGTTCT GTACGGGACCTGGAGGAGAAGC
KO330	To amplify p150-CC1	reverse	TCGTCATCCTTGTAATCTTAGAAATC AAAAGTCTCTGGCGGTGGCTG
KO331	To amplify pET28a backbone for p150-CC1	forward	TAAGATTACAAGGATGACGACGATA AGTAAGCGGC
KO332	To amplify pET28a backbone for p150-CC1	reverse	GGTCCTTGGAAGAGAACCTCAAGGG ATC
